# A Phenomenological Approach to Understanding the Association Between Psychosis and Violence

**DOI:** 10.1192/bjo.2022.166

**Published:** 2022-06-20

**Authors:** Coral Anderson, Rajan Nathan

**Affiliations:** 1 Cheshire and Wirral Partnership NHS Foundation Trust, Chester, United Kingdom; 2 University of Liverpool, Liverpool, United Kingdom; 3 John Moores University, Liverpool, United Kingdom; 4 University of Chester, Chester, United Kingdom

## Abstract

**Aims:**

Although there is an established but complex relationship between violence and psychosis, the nature of this relationship remains unclear. To date, there has been a predominant focus within group-level quantitative studies on specific types of psychopathology such as threat/control-override and command hallucinations. However, the literature has not produced a consensus on the profile of psychopathological predictors of violence. Furthermore, there is an emerging literature suggesting the predictive paradigm has limited clinical utility in the management of harm-related behaviour. In the way that phenomenological analysis has produced a fuller understanding of psychosis (that can inform improved aetiological and interventional frameworks), the authors assert that such an approach (with its focus on subjectivity) has the potential to advance our understanding of the relationship between psychosis and violence in a way that has clinical applicability. To test this assertion, it is necessary to develop a model of assessment and analysis. The aim of this paper is to develop an evidence-based model to explore the phenomenological underpinnings of violence in psychosis.

**Methods:**

A two-stage method was followed. Firstly, drawing on existing phenomenological accounts of psychosis and approaches to understanding the subjectivity of violence perpetration, the authors developed a pilot evaluation model. This was tested and revised by applying the model to phenomenological case reports of psychosis and violence.

**Results:**

The findings so far demonstrate that as well as the role of circumscribed psychopathology on the likelihood of violence, other experiences within the psychotic spectrum such as operative hyperreflexivity and disturbances of ipseity play an important role. Additionally, feelings of disconnectedness and loss of recognising others as real combined with impaired theory of mind can lead to loss of normal inhibitory processes and violent behaviour. In keeping with a recent shift in focus from strict diagnostic criteria to individual psychotic phenomena, existential analysis can be applied to explore changes in self-identity and sense of belonging in the world to develop our understanding of the association between psychosis and violence.

**Conclusion:**

The phenomenological model developed produces a fuller picture of the association between psychosis and violence. As such it may generate insight into the association between psychosis and violence that has greater clinical utility than existing psychopathology-based theories do. This needs to be evaluated by field testing of the approach.

